# 1-(2-Hy­droxy­eth­yl)-3-phenyl­thio­urea

**DOI:** 10.1107/S160053681201183X

**Published:** 2012-03-24

**Authors:** Antar A. Abdelhamid, Shaaban K. Mohamed, Mehmet Akkurt, Kuldip Singh, Herman Potgieter

**Affiliations:** a Chemistry and Environmental Science Division, School of Science & The Environment, Manchester Metropolitan University, M1 5GD, England; b Department of Physics, Faculty of Sciences, Erciyes University, 38039 Kayseri, Turkey; c Department of Chemistry, University of Leicester, Leicester, England; d School of Research, Enterprise & Innovation, Manchester Metropolitan University, M1 5GD, England

## Abstract

The title compound, C_9_H_12_N_2_OS, was obtained unexpectedly in a multicomponent reaction of an equimolar ratio of phenyl isothio­cyanate, malononitrile and amino­ethanol. The –C(H_2_)–N(H)–(C=S)–N(H)– methyl­thio­urea–methane group is almost normal to the phenyl ring, with a dihedral angle of 71.13 (9)°. The N—C—C—O torsion angle is 72.8 (2)°. In the crystal, mol­ecules are connected by N—H⋯O, O—H⋯S and N—H⋯O hydrogen bonds, forming a three-dimensional network.

## Related literature

For the biological activity of thio­ureas, see: Kilcigil & Altanlar (2006 ▶); Struga *et al.* (2007 ▶); Desai *et al.* (2007 ▶); Patel *et al.* (2007 ▶); Arslan *et al.* (2006 ▶); Katritzky & Gordeev (1991 ▶). For standard bond lengths, see: Allen *et al.* (1987 ▶). For hydrogen-bond motifs, see: Bernstein *et al.* (1995 ▶); Etter *et al.* (1990 ▶).
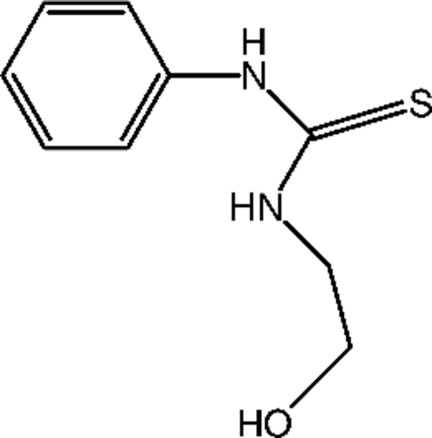


## Experimental

### 

#### Crystal data

C_9_H_12_N_2_OS
*M* *_r_* = 196.28
Tetragonal, 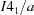

*a* = 26.170 (4) Å
*c* = 5.7775 (16) Å
*V* = 3956.8 (16) Å^3^
*Z* = 16
Mo *K*α radiation
μ = 0.29 mm^−1^
*T* = 150 K
0.27 × 0.09 × 0.08 mm

#### Data collection

Bruker APEX 2K CCD diffractometer
Absorption correction: multi-scan (*SADABS*; Sheldrick, 1996 ▶) *T*_min_ = 0.926, *T*_max_ = 0.977
15367 measured reflections
2056 independent reflections
1540 reflections with *I* > 2σ(*I*)
*R*_int_ = 0.095

#### Refinement

*R*[*F* ^2^ > 2σ(*F* ^2^)] = 0.048
*wR*(*F* ^2^) = 0.110
*S* = 0.98
2056 reflections
119 parameters
H-atom parameters constrained
Δρ_max_ = 0.31 e Å^−3^
Δρ_min_ = −0.24 e Å^−3^

### 

Data collection: *APEX2* (Bruker, 2005 ▶); cell refinement: *SAINT* (Bruker, 2005 ▶); data reduction: *SAINT*; program(s) used to solve structure: *SIR97* (Altomare *et al.*, 1999 ▶); program(s) used to refine structure: *SHELXL97* (Sheldrick, 2008 ▶); molecular graphics: *ORTEP-3* (Farrugia, 1997 ▶) and *PLATON* (Spek, 2009 ▶); software used to prepare material for publication: *WinGX* (Farrugia, 1999 ▶) and *PLATON*.

## Supplementary Material

Crystal structure: contains datablock(s) global, I. DOI: 10.1107/S160053681201183X/rk2342sup1.cif

Structure factors: contains datablock(s) I. DOI: 10.1107/S160053681201183X/rk2342Isup2.hkl

Supplementary material file. DOI: 10.1107/S160053681201183X/rk2342Isup3.cml

Additional supplementary materials:  crystallographic information; 3D view; checkCIF report

## Figures and Tables

**Table 1:** Hydrogen-bond geometry (Å, °)

*D*—H⋯*A*	*D*—H	H⋯*A*	*D*⋯*A*	*D*—H⋯*A*
N1—H1*A*⋯S1^i^	0.86	2.54	3.3676 (18)	163
O1—H1*B*⋯S1^ii^	0.82	2.40	3.2137 (18)	169
N2—H2*A*⋯O1^iii^	0.86	2.15	2.875 (2)	142

Symmetry codes: (i) 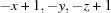; (ii) 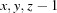; (iii) 
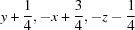
.

**Fig. 1.**
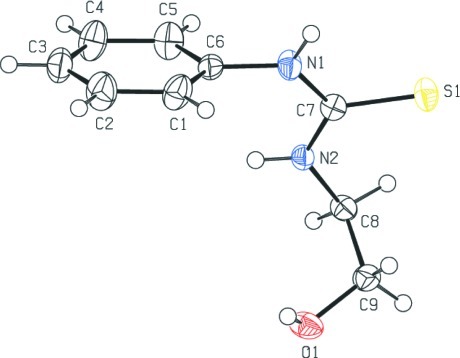
The molecular structure of I, showing the labelling of the non-H atoms. Displacement ellipsoids are drawn at the 50% probability level. H atoms are presented as a small spheres of arbitrary radius.

**Fig. 2.**
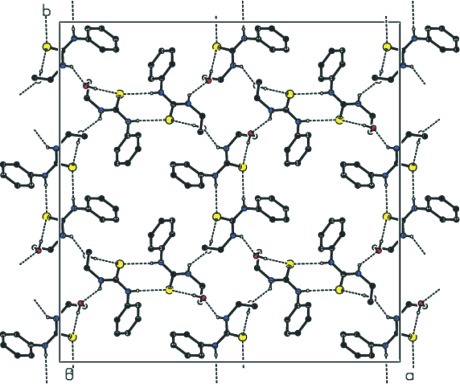
View of the crystal packing and hydrogen bonding of I down the a axis. H atoms not involved in hydrogen bonds have been omitted for clarity.

## supplementary crystallographic information

### Comment

Substituted thioureas are important organic compounds prompting the interest of chemists due to their broad spectrum of biological activities such as anti-*HIV*, antiviral, *HDL*-elevating, antibacterial, analgesic properties (Kilcigil & Altanlar, 2006; Struga *et al.*, 2007; Desai *et al.*, 2007; Patel *et al.*, 2007) and acting as fungicides (Arslan *et al.*, 2006). Industrially, thioureas act as corrosion inhibitors, antioxidant, and are polymer components (Katritzky & Gordeev, 1991). The title compound has been obtained as an unexpected product from our multicomponent reaction techniques of phenylisothiocyanate, malononitrile and amino ethanol under conventional heat.

In the title compound I (Fig. 1), the methylthiourea-methane group (–C8(H_2_)–N2(H)–(C7═S1)–N1(H)–) makes a dihedral angle of 71.13 (9)° with the C1-C6 phenyl ring. The N2–C8–C9–O1 torsion angle is 72.8 (2)°. The bond lengths and angles in I are in the normal range (Allen *et al.*, 1987).

Intramolecular C8–H8A···S1 contact help to stabilize the molecular conformation of I, generating a C(5) loop (Bernstein *et al.*, 1995; Etter *et al.*, 1990). The crystal packing is stabilized by N–H···O, O–H···S and N–H···O intermolecular hydrogen bonds, forming a three-dimensional network (Table 1, Fig. 2).

### Experimental

The 1-(2-hydroxyethyl)-3-phenylthiourea has been formed as an unexpected product from a multicomponent reaction of an equimolar ratio of phenylisothiocyanate, malononitrile and amino ethanol. The reaction mixture was heated at 374 K in dioxane (30 ml) for 3 h, then cooled at room temperature to afford a solid precipitate. The product was filtered off, washed with cold ethanol and recrystallized from ethanol. Colourless needles in a spiky shape have been isolated on slow evaporation of a diluted ethanol of the product (yield 39%, m.p. 393 K).

### Refinement

All H atoms were positioned geometrically and refined using a riding model with C–H = 0.93Å for aromatic, C–H = 0.97Å for methylene, O–H = 0.82Å for hydroxyl and N–H = 0.86Å for amine H atoms, and with *U*_iso_(H) = 1.2(1.5)*U*_eq_(C,N,O).

### Crystal data

**Table:**

C_9_H_12_N_2_OS	*D*_x_ = 1.318 Mg m^−^^3^
*M**_r_* = 196.28	Melting point: 393 K
Tetragonal, *I*4_1_/*a*	Mo *K*α radiation, λ = 0.71073 Å
Hall symbol: -I 4ad	Cell parameters from 800 reflections
*a* = 26.170 (4) Å	θ = 3.5–28.2°
*c* = 5.7775 (16) Å	µ = 0.29 mm^−^^1^
*V* = 3956.8 (16) Å^3^	*T* = 150 K
*Z* = 16	Needle, colourless
*F*(000) = 1664	0.27 × 0.09 × 0.08 mm

### Data collection

**Table:**

Bruker APEX 2K CCD diffractometer	2056 independent reflections
Radiation source: fine-focus sealed tube	1540 reflections with *I* > 2σ(*I*)
Graphite monochromator	*R*_int_ = 0.095
φ and ω scans	θ_max_ = 26.5°, θ_min_ = 1.6°
Absorption correction: multi-scan (*SADABS*; Sheldrick, 1996)	*h* = −32→32
*T*_min_ = 0.926, *T*_max_ = 0.977	*k* = −32→32
15367 measured reflections	*l* = −7→7

### Refinement

**Table:**

Refinement on *F*^2^	Primary atom site location: structure-invariant direct methods
Least-squares matrix: full	Secondary atom site location: difference Fourier map
*R*[*F*^2^ > 2σ(*F*^2^)] = 0.048	Hydrogen site location: inferred from neighbouring sites
*wR*(*F*^2^) = 0.110	H-atom parameters constrained
*S* = 0.98	*w* = 1/[σ^2^(*F*_o_^2^) + (0.0501*P*)^2^] where *P* = (*F*_o_^2^ + 2*F*_c_^2^)/3
2056 reflections	(Δ/σ)_max_ = 0.001
119 parameters	Δρ_max_ = 0.31 e Å^−^^3^
0 restraints	Δρ_min_ = −0.24 e Å^−^^3^

### Special details

**Table:**

Geometry. Bond distances, angles *etc*. have been calculated using the rounded fractional coordinates. All s.u.'s are estimated from the variances of the (full) variance-covariance matrix. The cell s.u.'s are taken into account in the estimation of distances, angles and torsion angles.
Refinement. Refinement on *F*^2^ for ALL reflections except those flagged by the user for potential systematic errors. Weighted *R*-factors *wR* and all goodnesses of fit *S* are based on *F*^2^, conventional *R*-factors *R* are based on *F*, with *F* set to zero for negative *F*^2^. The observed criterion of *F*^2^ > σ(*F*^2^) is used only for calculating *R*-factor *etc*. and is not relevant to the choice of reflections for refinement. *R*-factors based on *F*^2^ are statistically about twice as large as those based on *F*, and *R*-factors based on ALL data will be even larger.

### Fractional atomic coordinates and isotropic or equivalent isotropic displacement parameters (Å^2^)

**Table:**

	*x*	*y*	*z*	*U*_iso_*/*U*_eq_	
S1	0.53773 (2)	0.07294 (2)	0.52460 (10)	0.0281 (2)	
O1	0.56205 (6)	0.17228 (6)	−0.1675 (3)	0.0322 (5)	
N1	0.45696 (7)	0.04810 (6)	0.2790 (3)	0.0269 (6)	
N2	0.49000 (6)	0.12770 (6)	0.1987 (3)	0.0215 (5)	
C1	0.41711 (9)	0.01902 (9)	−0.0757 (4)	0.0352 (8)	
C2	0.37649 (9)	0.01894 (10)	−0.2287 (4)	0.0416 (9)	
C3	0.33488 (9)	0.04979 (9)	−0.1906 (4)	0.0365 (8)	
C4	0.33371 (9)	0.08094 (10)	−0.0010 (4)	0.0394 (9)	
C5	0.37390 (9)	0.08096 (9)	0.1544 (4)	0.0346 (8)	
C6	0.41558 (8)	0.05020 (8)	0.1157 (4)	0.0237 (7)	
C7	0.49204 (8)	0.08449 (8)	0.3204 (4)	0.0216 (7)	
C8	0.52638 (8)	0.16964 (8)	0.2207 (4)	0.0231 (7)	
C9	0.57332 (8)	0.16375 (9)	0.0697 (4)	0.0282 (7)	
H1	0.44530	−0.00190	−0.10200	0.0420*	
H1A	0.45960	0.02050	0.35900	0.0320*	
H1B	0.55420	0.14510	−0.22890	0.0480*	
H2	0.37740	−0.00210	−0.35820	0.0500*	
H2A	0.46550	0.13110	0.10050	0.0260*	
H3	0.30760	0.04950	−0.29360	0.0440*	
H4	0.30580	0.10220	0.02370	0.0470*	
H5	0.37270	0.10170	0.28490	0.0410*	
H8A	0.53710	0.17210	0.38100	0.0280*	
H8B	0.50930	0.20130	0.18060	0.0280*	
H9A	0.59920	0.18790	0.11950	0.0340*	
H9B	0.58700	0.12960	0.08840	0.0340*	

### Atomic displacement parameters (Å^2^)

**Table:**

	*U*^11^	*U*^22^	*U*^33^	*U*^12^	*U*^13^	*U*^23^
S1	0.0314 (3)	0.0206 (3)	0.0322 (3)	−0.0001 (2)	−0.0121 (3)	0.0010 (2)
O1	0.0448 (10)	0.0284 (9)	0.0234 (9)	−0.0100 (8)	0.0032 (8)	−0.0016 (7)
N1	0.0285 (10)	0.0191 (9)	0.0332 (11)	−0.0045 (8)	−0.0107 (8)	0.0074 (8)
N2	0.0214 (9)	0.0219 (9)	0.0211 (10)	−0.0023 (7)	−0.0036 (7)	0.0015 (7)
C1	0.0253 (12)	0.0417 (15)	0.0385 (14)	0.0047 (11)	−0.0008 (11)	−0.0120 (12)
C2	0.0388 (15)	0.0516 (16)	0.0343 (15)	0.0003 (12)	−0.0058 (12)	−0.0151 (12)
C3	0.0292 (13)	0.0402 (15)	0.0402 (15)	−0.0034 (11)	−0.0118 (11)	0.0027 (12)
C4	0.0291 (13)	0.0384 (14)	0.0508 (17)	0.0090 (11)	−0.0089 (12)	−0.0073 (12)
C5	0.0362 (14)	0.0298 (13)	0.0377 (15)	0.0049 (11)	−0.0063 (11)	−0.0102 (11)
C6	0.0233 (11)	0.0201 (11)	0.0278 (12)	−0.0053 (9)	−0.0038 (9)	0.0052 (9)
C7	0.0233 (11)	0.0191 (11)	0.0223 (12)	0.0014 (9)	0.0007 (9)	−0.0033 (9)
C8	0.0291 (12)	0.0188 (11)	0.0214 (12)	−0.0039 (9)	−0.0015 (9)	−0.0001 (9)
C9	0.0267 (12)	0.0325 (13)	0.0255 (13)	−0.0068 (10)	−0.0002 (10)	−0.0011 (10)

### Geometric parameters (Å, º)

**Table:**

S1—C7	1.707 (2)	C4—C5	1.383 (3)
O1—C9	1.420 (3)	C5—C6	1.374 (3)
O1—H1B	0.8200	C8—C9	1.515 (3)
N1—C6	1.437 (3)	C1—H1	0.9300
N1—C7	1.344 (3)	C2—H2	0.9300
N2—C7	1.333 (3)	C3—H3	0.9300
N2—C8	1.459 (3)	C4—H4	0.9300
N1—H1A	0.8600	C5—H5	0.9300
N2—H2A	0.8600	C8—H8A	0.9700
C1—C2	1.383 (3)	C8—H8B	0.9700
C1—C6	1.375 (3)	C9—H9A	0.9700
C2—C3	1.373 (3)	C9—H9B	0.9700
C3—C4	1.366 (3)		
			
C9—O1—H1B	109.00	C2—C1—H1	120.00
C6—N1—C7	127.18 (17)	C6—C1—H1	120.00
C7—N2—C8	124.50 (17)	C1—C2—H2	120.00
C6—N1—H1A	116.00	C3—C2—H2	120.00
C7—N1—H1A	116.00	C2—C3—H3	120.00
C8—N2—H2A	118.00	C4—C3—H3	120.00
C7—N2—H2A	118.00	C3—C4—H4	120.00
C2—C1—C6	119.5 (2)	C5—C4—H4	120.00
C1—C2—C3	120.4 (2)	C4—C5—H5	120.00
C2—C3—C4	119.8 (2)	C6—C5—H5	120.00
C3—C4—C5	120.3 (2)	N2—C8—H8A	109.00
C4—C5—C6	119.9 (2)	N2—C8—H8B	109.00
N1—C6—C1	118.90 (19)	C9—C8—H8A	109.00
C1—C6—C5	120.1 (2)	C9—C8—H8B	109.00
N1—C6—C5	120.9 (2)	H8A—C8—H8B	108.00
S1—C7—N1	118.42 (16)	O1—C9—H9A	109.00
N1—C7—N2	118.68 (19)	O1—C9—H9B	109.00
S1—C7—N2	122.89 (16)	C8—C9—H9A	109.00
N2—C8—C9	113.75 (18)	C8—C9—H9B	109.00
O1—C9—C8	111.81 (17)	H9A—C9—H9B	108.00
			
C6—N1—C7—S1	179.93 (17)	C2—C1—C6—N1	177.1 (2)
C7—N1—C6—C1	111.2 (3)	C2—C1—C6—C5	0.1 (3)
C7—N1—C6—C5	−71.9 (3)	C1—C2—C3—C4	0.3 (4)
C6—N1—C7—N2	−0.8 (3)	C2—C3—C4—C5	−1.0 (4)
C7—N2—C8—C9	86.7 (3)	C3—C4—C5—C6	1.2 (4)
C8—N2—C7—S1	1.1 (3)	C4—C5—C6—N1	−177.6 (2)
C8—N2—C7—N1	−178.12 (19)	C4—C5—C6—C1	−0.7 (3)
C6—C1—C2—C3	0.1 (4)	N2—C8—C9—O1	72.8 (2)

### Hydrogen-bond geometry (Å, º)

**Table:**

*D*—H···*A*	*D*—H	H···*A*	*D*···*A*	*D*—H···*A*
N1—H1*A*···S1^i^	0.86	2.54	3.3676 (18)	163
O1—H1*B*···S1^ii^	0.82	2.40	3.2137 (18)	169
N2—H2*A*···O1^iii^	0.86	2.15	2.875 (2)	142
C8—H8*A*···S1	0.97	2.72	3.094 (2)	103

Symmetry codes: (i) −*x*+1, −*y*, −*z*+1; (ii) *x*, *y*, *z*−1; (iii) *y*+1/4, −*x*+3/4, −*z*−1/4.
